# Construction and Characterization of a Recombinant Human Respiratory Syncytial Virus Encoding Enhanced Green Fluorescence Protein for Antiviral Drug Screening Assay

**DOI:** 10.1155/2018/8431243

**Published:** 2018-01-15

**Authors:** Min Xu, Yue-Ying Jiao, Yuan-Hui Fu, Nan Jiang, Yuan-Bo Zheng, Yi-Fei Yan, Mei Zhang, Yan-Peng Zheng, Wu-Yang Zhu, Xiang-Lei Peng, Jin-Sheng He

**Affiliations:** ^1^College of Life Sciences and Bioengineering, Beijing Jiaotong University, Beijing 100044, China; ^2^Department of Immunology, Anhui Medical University, Hefei 230032, China; ^3^National Institute for Viral Disease Control and Prevention, Chinese Center for Disease Control and Prevention, Beijing 102206, China

## Abstract

Human respiratory syncytial virus (RSV) is the single most important cause of lower respiratory tract disease in infants and young children and a major viral agent responsible for respiratory tract disease in immunosuppressed individuals and the elderly, but no vaccines and antiviral drugs are available. Herein the recombinant RSV (rRSV) encoding enhanced green fluorescence protein (EGFP, rRSV-EGFP) was constructed and the potential for screening anti-RSV drugs was investigated. The recombinant plasmid of pBRATm-rRSV-EGFP, containing T7 transcription cassette composed of T7 promoter, RSV antigenomic cDNA with EGFP gene, HDV ribozyme (*δ*), and T7 terminator in the order of 5′ to 3′, was constructed and cotransfected into BHK/T7-9 cells together with helper plasmids encoding N, P, L, and M2-1 gene, respectively. The rescued rRSV-EGFP was confirmed by increasing expression of EGFP over blind passages and by RT-PCR. rRSV-EGFP was comparable to the other two recombinant RSVs encoding red fluorescent protein (RFP, rRSV-RFP) or luciferase (Luc, rRSV-Luc) in the growth kinetic, and there was a difference in sensitivity between them for screening anti-RSV agents based on infection of HEp-2 cells. The EGFP-encoding rRSV has been constructed and rescued successfully and has the potential for high-throughput anti-RSV drug screening in vitro.

## 1. Introduction

Human respiratory syncytial virus (RSV) is a human respiratory tract pathogen of the Mononegavirales in the* Orthopneumovirus* genus within the Pneumoviridae family [1]. The virus causes over 30 million acute lower respiratory tract illnesses annually in children and is also an important cause of infant pneumonia mortality worldwide [2]. There is currently no licensed vaccine available to prevent RSV infection, and anti-RSV treatments are far from being satisfying. RSV F monoclonal antibody of palivizumab is effective in preventive treatment for high-risk infants, but it is costly and has no definite role in therapy for established infection; ribavirin, the only licensed antiviral agent for the treatment of RSV infection, is not recommended to be used routinely unless in patients with severe low respiratory tract disease due to its efficacy and toxicity issues [3]. Therefore, developing novel anti-RSV drugs becomes very urgent.

Searching RSV inhibitors is generally performed by laborious, time-consuming, and expensive methods. For example, the methods in use include immunoplaque upon enzyme immunoassay, plaque upon cytopathic effect (CPE), quantitative reverse transcription polymerase chain reaction- (qRT-) PCR, and enzyme-linked immunosorbent assays (ELISA). In contrast, the application of recombinant RSV virus (rRSV) encoding reporter genes such as enhanced green fluorescent protein (EGFP, rRSV-EGFP) and luciferase (Luc) would be more practicable to screen RSV inhibitors, even available for high-throughput screening (HTS) when combined with microplates and automated plate readers [4–7].

Here, firstly, we described the construction of a recombinant RSV virus, rRSV-EGFP, based on reverse genetic techniques. The full-length RSV Long antigenomic cDNA carrying EGFP expression cassette, flanked by T7 promoter, HDV ribozyme and T7 terminator, was cloned and placed into cloning vector derived from pBR322. After the resulting recombinant plasmid and the helper plasmids encoding nucleoprotein (N), phosphoprotein (P), large protein (L), and M2 ORF protein 1 (M2-1), respectively, were cotransfected into BHK/T7-9 cells expressing the T7 RNA polymerase, the recombinant virus rRSV-EGFP was successfully rescued and characterized in vitro. Then the growing kinetic of the resulting rRSV-EGFP and its feasibility for selecting RSV inhibitors in HEp-2 cells were investigated. Compared with rRSV-RFP and rRSV-Luc encoding red fluorescence protein (RFP) and Luc, respectively, and wild-type RSV Long strain (*wt*RSV Long), rRSV-EGFP displayed the potential for high-throughput anti-RSV compound screening in vitro.

## 2. Materials and Methods

### 2.1. Cells and Viruses

BHK/T7-9 cells (baby hamster kidney cells expressing T7 RNA polymerase) were kindly provided by Professor W. Y. Zhu, CDC, Beijing, China, and maintained in DMEM (Gibco BRL, Gaithersburg, USA) with 10% of tryptose phosphate broth (Sigma, Darmstadt, Germany), 5% of fetal bovine serum (FBS, Hyclone, Logan, USA), 2 mmol/L L-glutamine (Amresco, Solon, USA), and antibiotics (100 IU/ml penicillin, 10 *μ*g/ml streptomycin, and 0.25 *μ*g/ml amphotericin B), as well as hygromycin (600 ng/ml, Sigma) [8]. HEp-2 cells, Vero cells, and 293T cells (ATCC, Rockefeller, MD, USA) were grown in DMEM containing 2 mmol/L L-glutamine and 10% FBS. Subgroup A* wt*RSV Long (ATCC, Rockefeller, MD, USA), rRSV-RFP, and rRSV-Luc (kindly provided by Dr. Marie-Anne Rameix-Welti, Unite de Virologie et Immunologie Moleculaires, Paris, France) were propagated in HEp-2 cells in DMEM supplemented with 2% FBS, L-glutamine (2 mmol/L), and antibiotics (40 IU/ml penicillin G and 100 *μ*g/ml streptomycin) and 0.2% sodium bicarbonate.* wt*RSV Long and recombinant RSVs were purified by ultracentrifugation and titrated for infectivity by immunoplaque assay [9]. Briefly, 10-fold serial dilution of 100 *μ*l RSV sample was absorbed onto 80% confluency of HEp-2 cells in 96-well plate in triplicate for 1 h at 37°C, and then the media were removed and the cells were rinsed with DMEM without FBS before adding DMEM containing 0.9% methyl cellulose (Sigma). After 3 days of incubation at 37°C under 5% CO_2_, the monolayer was fixed with 95% cold alcohol and viral replication on the monolayer was revealed by goat anti-RSV antibody (Millipore, Billerica, MA, USA), incubated with horseradish peroxidase rabbit anti-goat IgG (Santa Cruz Biotechnology, California, CA, USA), and then visualized after adding TMB (Promega, Madison, USA). RSV titers were expressed as plaque-forming units per ml (pfu/ml). All experiments with infectious virus were performed in the BSL-2 Laboratory at the Beijing Jiaotong University, Beijing, China.

### 2.2. Plasmids

The cloning vector pBRATm, originally from vector pBR322 with modifications such as the optimized multiple cloning site (MCS), was preserved in our lab, and the helper plasmids of pCITE-N, pCITE-P, pCITE-L, and pCITE-M2-1 encoding N, P, L, and M2-1 proteins, respectively, were kindly provided by Dr. Marie-Anne Rameix-Welti.

### 2.3. Construction of rRSV-EGFP Antigenomic cDNA

**Figure 1:**
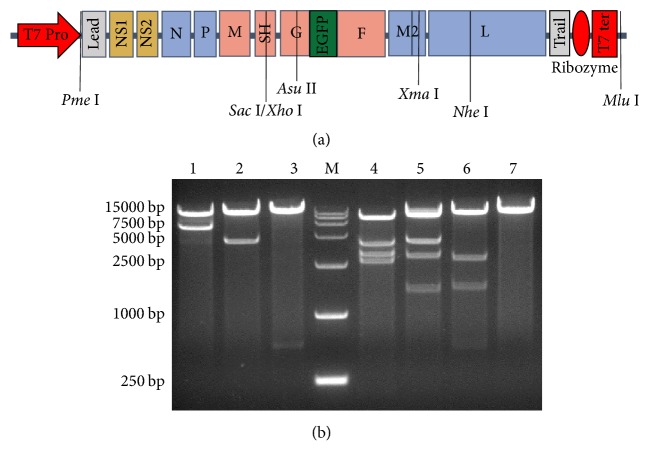
The schematic diagram and identification of pBRATm-rRSV-EGFP. (a) The recombinant full-length RSV antigenomic cDNA cloned from wide-type RSV Long strain with the inserted EGFP gene expression cassette in the position between G and F and flanked by T7 promoter and HDV ribozyme-T7 terminator in the 5′ and 3′ ends, respectively. T7 pro: T7 promoter; T7 ter: T7 terminator; Lead: Leader; Trail: Trailer; Ribozyme: HDV ribozyme. (b) Identification of pBRATm-RSV-EGFP by restriction endonuclease analysis. M, DNA Ladder DL15000; pBRATm-RSV-EGFP was digested with* Asu *II and* Nhe* I (1),* Pme* I and* Xho* I (2),* Asu* II and* Sac* I (3),* Eco*R V (4),* Spe* I (5),* Afl* II (6), or* Mlu* I (7).

To construct antigenomic cDNA of rRSV-EGFP, a series of cloning steps from cDNA segments, synthesized either by RT-PCR from intracellular RSV mRNA or by virus genomic RNA- (vRNA-) derived complementary RNA (cRNA), or by overlapping PCR, were performed. The gene order in the resulting antigenomic cDNA of rRSV-EGFP was as follows, 5′-leader-NS1-NS2-N-P-M-SH-G-EGFP-F-M2-1/M2-2-L-trailer-3′, and flanked by T7 promoter in the 5′ end and by HDV ribozyme-T7 terminator in the 3′ end, respectively (Figure 1(a)). Then, the full-length cDNA of rRSV-EGFP antigenome was cloned into the sites of* Pme* I and* Mlu* I, encompassed by the transcription terminator signals of vaccinia virus, in pBRATm, and the ensuing plasmid was named pBRATm-RSV-EGFP.

### 2.4. Recovery of rRSV-EGFP

Monolayer culture of BHK/T7-9 cells, grown overnight in a six-well dish, was cotransfected with both pBRATm-RSV-EGFP and four helper plasmids (pCITE-N, pCITE-P, pCITE-L, and pCITE-M2-1) by Lipofectamine 2000 (Invitrogen, CA, USA). For each well of cells to be transfected, the transfection mixtures were prepared as follows: pBRATm-RSV-EGFP 1.25 *μ*g, pCITE-N 1 *μ*g, pCITE-P 1 *μ*g, pCITE-L 0.5 *μ*g, and pCITE-M2-1 0.25 *μ*g. After mixing the above plasmids with the Lipofectamine 2000 together gently and incubating for 20 min at room temperature, the mixtures of DNA-lipo-OptiMEM (Gibco) were added to each well of the cells. The cells were incubated at 37°C in a 5% CO_2_ incubator and observed the green fluorescence under the inverted fluorescence microscope daily (NIKON, Japan). For the blind passage, HEp-2 cells were adsorbed with 400 *μ*l of the suspension from the harvested cotransfected cells at 33°C for 2 h. Then the infected HEp-2 cells were rinsed by PBS before adding DMEM with 2% FBS and incubated at 37°C and monitored under the fluorescence microscope every day.

### 2.5. Characterization of rRSV-EGFP

Serial passages from passage 1 (p1) to p9 were performed in HEp-2 cells and observed under fluorescence microscope. The rescued recombinant virus was also assayed by RT-PCR using GoScript™ RT-PCR kit (Promega) to amplify the mRNA transcript from the encoded EGFP (forward primer, 5′-GTCTCCACAACATCCGAGCACCCATC-3′; reverse primer, 5′-CTGCAGCGAGGATTGT GGTAATTGC-3′) by rRSV-EGFP. Meanwhile, the infectivity of the rRSV-EGFP was titrated with the method of immunoplaque assay as described previously [9]. For growth kinetic assay, it was performed as reported previously [10]. Briefly, HEp-2 cells were infected with* wt*RSV Long, rRSV-RFP, or rRSV-Luc at the multiplicity of infection (MOI) of 0.1 in triplicate and incubated at 37°C. Viruses were harvested every other 24 h postinfection, and virus titers were determined by immunoplaque assay as described previously [9]. For the replication capacity of the rRSV-EGFP in different cells, HEp-2, Vero, or 293T cells were infected with 0.1 MOI of rRSV-EGFP and incubated at 37°C. Viruses were harvested at 48 h postinfection and titers were determined in these cell lines individually with the mentioned immunoplaque assay above and quantitative real-time PCR (RT-qPCR). RT-qPCR was performed to determine RSV replication as reported previously [11]. Briefly, samples of viral RNA were isolated from infected cells using Trizol reagent (Invitrogen) according to the manufacturer's instructions. 1.5 *μ*g RNA samples were reverse transcribed with the primer of oligo dT and at 70°C 5 min, and the cDNA was further synthesized 5 min on ice, then 5 min at 20°C, 1 h at 42°C, and 15 min at 70°C and then stored at 4°C. The qPCR was performed by using SYBR green probe (Tiangen Biotech Co., Ltd. Beijing, China). The primers for the RSV nucleoprotein (N) gene were as follows: forward primer, 5′-AGATCAACTTCTGTCATCCAGCAA-3′; reverse primer, 5′-GCACATCATAATTAGGAGTATCAAT-3′ [12]. Thermal cycling conditions included 15 min at 95°C, followed by 45 cycles of 15 s at 95°C and 1 min at 60°C. The specificity of the qPCR products was verified by melting point analysis from 45°C to 95°C.

### 2.6. Large Scale of Preparation and Purification of Viruses

The viral samples were inoculated onto HEp-2 cells with 70%–80% confluence and incubated for 3 days at 37°C under 5% CO_2_. After the syncytia formation, the cells were scraped off and centrifuged at 3000 rpm for 10 min at 4°C to remove cellular debris. Supernatants were pooled, filtered through a 0.45 *μ*m sterile filter (Merck Millipore, Carolina, USA) and purified by ultracentrifugation on sucrose cushion gradient (10% sucrose, Sigma) at 17,000 rpm in P28S rotor (Hitachi, Japan) for 2 h at 4°C. The pellet was suspended in 300 *μ*l 10% sucrose and divided into aliquots and stored at −80°C. The infectivity of the RSV was titrated using the method of immunoplaque assay as described previously [9].

### 2.7. The Feasibility of Screening Antiviral Agents Based on rRSV-EGFP

HEp-2 cells were infected in triplicate with either rRSV-EGFP, rRSV-RFP, or rRSV-Luc in 0.1 MOI and treated with either 0.5 *μ*mol/L compound P13 [13], 10 *μ*mol/L ribavirin [14], 0.4 *μ*mol/L mycophenolic acid [15], or 1.5 *μ*mol/L dequalinium chloride [16]. The doses for the employed antiviral agents were each determined upon the references above or the preexperiment performed in our lab. Following incubation at 37°C for 48 h, rRSV-EGFP- or rRSV-RFP-infected cells were observed with inverted fluorescence microscope and quantitatively measured by multifunctional microplate reader (SpectraMax M5e, Molecular Devices, Sunnyvale, USA) with excitation and emission wavelengths of 479 and 517 nm (EGFP), 580 and 620 nm (RFP), respectively, and expressed as relative fluorescence units (RFU). rRSV-Luc-infected cells were incubated at 37°C for 48 h, added with 50 *μ*l mixture (1 mL buffer mixed with 10 *μ*l substrate) per well with gentle shaking, and quantitatively measured using chemiluminescence multifunctional microplate reader (LUMIstarOPTIMA, BMG LATECH, Germany), and expressed as relative light units (RLU) [4]. For the titration assay, the methods of immunoplaque and RT-qPCR mentioned above were in use.

### 2.8. Statistical Analyses

Statistical analyses of data were performed using GraphPad Prism 5 software (GraphPad Software, La Jolla, CA). Comparison of differences was conducted using an independent, two-sided Student's* t*-test. *P* < 0.05 was considered statistically significant.

## 3. Results and Discussion

### 3.1. Cloning and Identification of Full-Length Antigenomic cDNA Encoding rRSV-EGFP

The recombinant full-length cDNA encoding rRSV-EGFP was obtained by stepwise assembly of the cloned cDNA segments, and the EGFP gene expression cassette was inserted in the position between G and F as shown in Figure 1(a). The resulting pBRATm-RSV-EGFP encoding the full-length antigenomic cDNA of rRSV-EGFP was expected to be 19543 bp in size and subjected to analyze by restriction endonucleases. The resulting fragment sizes and patterns were consistent with the anticipated, as shown in Figure 1(b). Then, it was further confirmed by DNA sequencing (data not shown). The nucleotide sequence of the full-length antigenome cDNA of rRSV-EGFP was the same as that of* wt*RSV Long in GenBank (AY911262.1), with the exception of the deleted 112 bp in the noncoding region following the SH gene and several single-nucleotide substitution mutations for the stability of RSV antigenome cDNA [17] as well as the added EGFP coding gene.

### 3.2. Recovery of rRSV-EGFP

**Figure 2:**
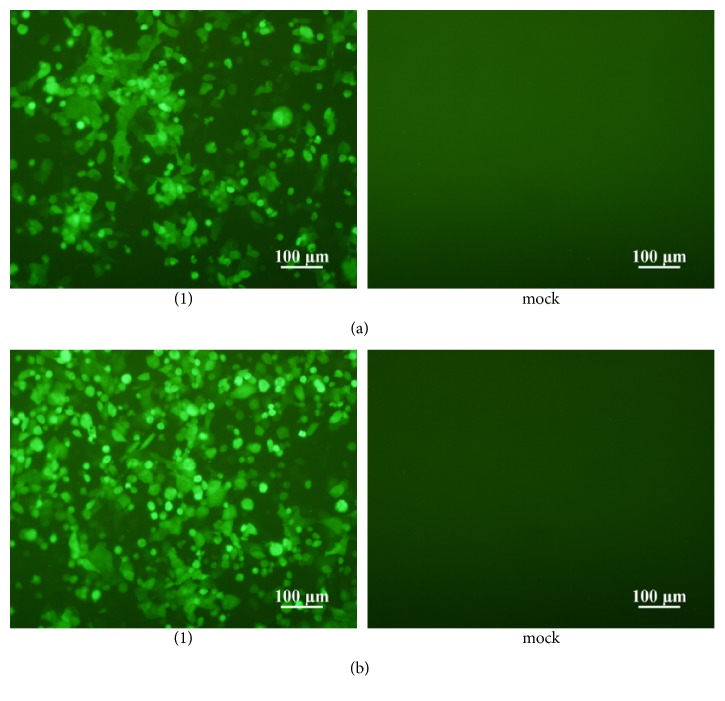
Recovery of rRSV-EGFP. (a) BHK-T7/9 cells cotransfected with pBRATm-RSV-EGFP and four helper plasmids (1) or cotransfected with only four helper plasmids (mock). (b) The suspensions from BHK-T7/9 cells cotransfected by pBRATm-RSV-EGFP and four helper plasmids (1), or only by four helper plasmids (mock) inoculated into HEp-2 cells for the blind passage. The fluorescence signals were observed around 120 h and about 72 h under the inverted fluorescence microscope following the posttransfection and the first blind passage, respectively.

To generate mature virions of rRSV-EGFP, plasmids encoding RSV antigenome cDNA, and helper proteins were cotransfected together to BHK/T7-9 cells. Following this performance, the fluorescence signals from the successfully rescued rRSV-EGFP appeared in the BHK/T7-9 cells around 120 h posttransfection and increased in the HEp-2 cells about 72 h after the first blind passage, as shown in Figures 2(a) and 2(b). These results demonstrated that we successfully rescued the recombinant virus carrying the EGFP gene and that the recovered rRSV-EGFP replicated pretty well in the susceptible cells.

### 3.3. Characterization of rRSV-EGFP

**Figure 3:**
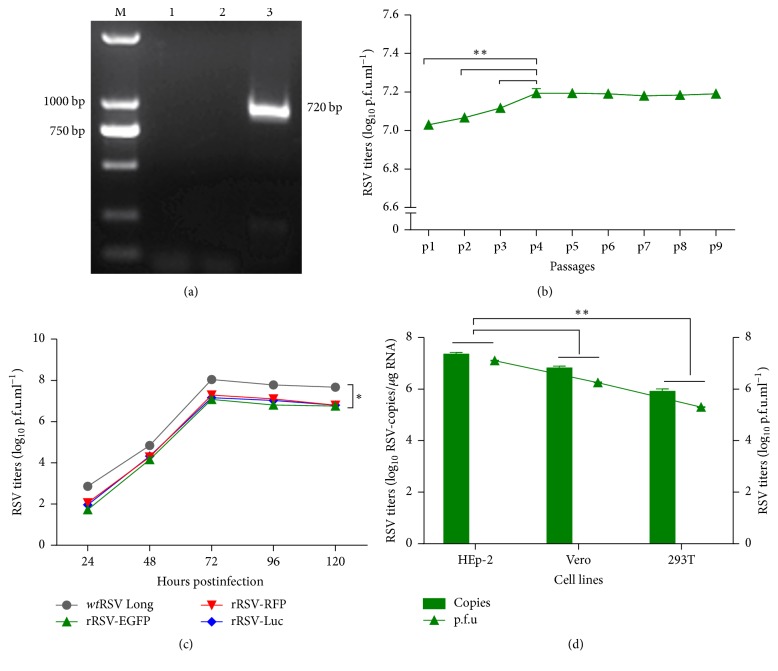
Characterization of rRSV-EGFP. (a) Identification of the rescued rRSV-EGFP by RT-PCR. M, DNA Ladder DL2000; control of HEp-2 cells (1),* wt*RSV Long infected HEp-2 cells (2), or rRSV-EGFP infected HEp-2 cells (3). (b) The replication titers of rRSV-EGFP during serial blind passages from p1 to p9 by immunoplaque assay. (c) The growth kinetic of rRSV-EGFP. The growth curve for rRSV-EGFP was compared with those for* wt*RSV Long, rRSV-RFP, and rRSV-Luc. Each virus was harvested every other 24 h postinfection and titers were assayed by immunoplaque assay. (d) The replication capacity of the rRSV-EGFP in HEp-2, Vero, or 293T cells. Viruses were harvested at 48 h postinfection and titers were determined by assays of immunoplaque and RT-qPCR. Data were shown as mean ± SD. ^*∗*^*P* < 0.05, ^*∗∗*^*P* < 0.01.

RT-PCR assay showed that the EGFP fragment, about 720 bp, was only detected in the cell samples infected by the rescued rRSV-EGFP other than by* wt*RSV Long as shown in Figure 3(a). The titers of rRSV-EGFP increased rapidly during passages from passage 1 (p1) to p4 (*P* < 0.05) in HEp-2 cells through the titration assay by immunoplaque method and remained constant following P4 in Figure 3(b). To further characterize rRSV-EGFP, the growth kinetics were also evaluated and compared in HEp-2 cells infected by viruses. The growth reached plateau at 72 h postinfection of HEp-2 cells for all the viruses and at the titer of 1.0 × 10^8^ pfu ml^−1^ for* wt*RSV Long, compared with 1.1 × 10^7^ pfu ml^−1^ for rRSV-EGFP, 2.0 × 10^7^ pfu ml^−1^ for rRSV-RFP and 1.5 × 10^7^ pfu ml^−1^ for rRSV-Luc as shown in Figure 3(c). The approximately 10-fold lower replication capability than the* wt*RSV Long indicated the inserted reporter genes attenuated the multiplication of the corresponding recombinant virus to a similar extent, although EGFP and the other reporter proteins have been known few toxicity or inhibitory activity on the cells or viruses. The replication capacity of the rRSV-EGFP in each cell line of HEp-2, Vero, and 293T was also determined and compared by immunoplaque assay and RT-qPCR, and the results showed that rRSV-EGFP could infect and grow in HEp-2 cells more efficiently (Figure 3(d)).

### 3.4. The Feasibility of Screening Antiviral Agents Based on rRSV-Infected HEp-2 Cells

In order to verify the feasibility to select anti-RSV drugs by rRSV-EGFP, we did the test analysis with the known RSV inhibitors including ribavirin and compound P13, as well as two potential anti-RSV compounds of mycophenolic acid and dequalinium chloride. Then, we evaluated and compared the inhibitory activity of these antiviral agents on rRSV-EGFP, rRSV-RFP, rRSV-Luc, or* wt*RSV Long by the reduced replication in HEp-2 cells based on assays both qualitatively and quantitatively. These analyses include the fluorescence signals observed from the encoded EGFP in rRSV-EGFP, or from the encoded RFP in rRSV-RFP under fluorescence microscope, virus titration assayed by immunoplaque, and RT-qPCR, as well as fluorescence or luminescence intensities calculated upon multifunctional microplate reader following treatments with ribavirin, P13, mycophenolic acid, or dequalinium chloride.

**Figure 4:**
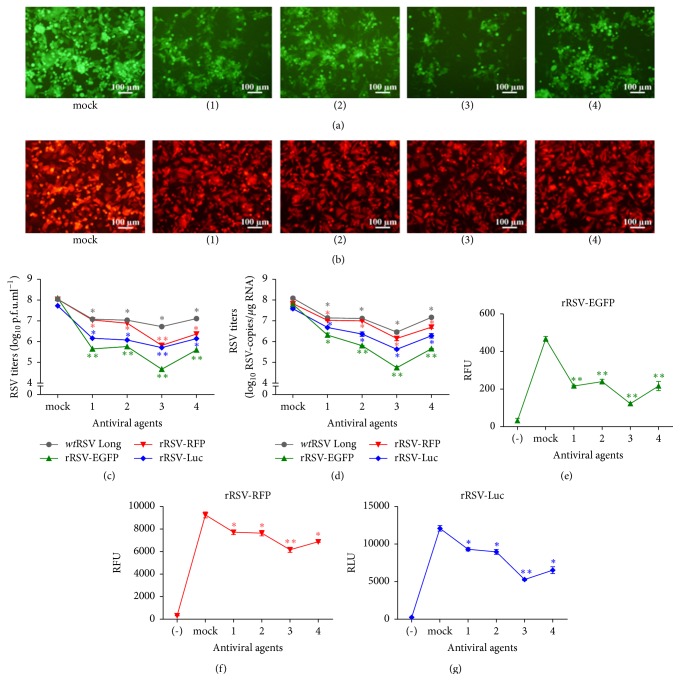
Inhibitive activities of antiviral agents on the replication of rRSV-EGFP in HEp-2 cells determined and compared with rRSV-RFP, rRSV-Luc, and* wt*RSV Long. The fluorescence signals from rRSV-EGFP or rRSV-RFP were observed under invert fluorescence microscope (a-b), the titers from rRSV-EGFP, rRSV-RFP, rRSV-Luc, or* wt*RSV Long were analyzed by immunoplaque and RT-qPCR (c-d), and the intensities of fluorescence or luminescence from rRSV-EGFP, rRSV-RFP, or rRSV-Luc were detected by multifunctional microplate reader and expressed as relative fluorescence units (RFU) and relative light units (RLU), respectively (e–g), following treatments with 10 *μ*mol/L ribavirin (1), 0.5 *μ*mol/L compound P13 (2), 0.4 *μ*mol/L mycophenolic acid (3), 1.5 *μ*mol/L dequalinium chloride (4), or no antiviral agent (mock) for 48 h. And HEp-2 cells were used as control of background levels (-). Data were shown as mean ± SD. ^*∗*^*P* < 0.05, ^*∗∗*^*P* < 0.01.

The results from fluorescence observed under fluorescence microscope (Figures 4(a) and 4(b)) showed the cells infected by rRSV-EGFP rather than rRSV-RFP displayed significant decline after treating with all of the antiviral agents compared to the mock-treated group. As for the virus titration assay, the titers of* wt*RSV Long, rRSV-EGFP, rRSV-RFP, and rRSV-Luc all reduced significantly following treatments with each of the antiviral agents, but the most evident decline arose in rRSV-EGFP, compared to mock-treated group as shown in Figures 4(c) and 4(d). The results from the assay of quantitative fluorescence intensities as shown in Figures 4(e)–4(g) exhibited approximately the same tendency to the data from titration assay. Taken together, these data showed that the rRSV-EGFP displayed better sensitivity in the assay of RSV inhibition activity from the tested antiviral agents and at the indicated doses when compared with the other two recombinant RSVs expressing RFP or Luc.

The rRSV-EGFP was constructed from the same parent virus strain,* wt*RSV Long, as the other two recombinant RSVs, except for the difference in the inserted position of the reporter genes. Instead of being located between P and M genes as RFP and Luc [4], EGFP gene was positioned between G and F genes. However, the location of the reporter genes has little influence on their growth kinetics in terms of the observed maximal growth rate and peak titers in Figure 3(c). Therefore, the distinct technique employed for each reporter signal acquisition and processing may be the most likely explanation for the altered sensitivity to these test inhibitors against RSV.

Additionally, an HTS method for antiviral compound based on EGFP-expressing recombinant RSV was indeed introduced in principle by Kwanten et al. [5]. However, the data on optimal parameters for the assay are still needed to be explored. In conclusion, although the mechanism underlying this observation needs to be further investigated, our results suggest that rRSV-EGFP has the potential for high-throughput anti-RSV drug screening in vitro following the specifications to be set.

## 4. Conclusion

In summary, the results show that we have successfully recovered rRSV-EGFP, the replication of which is sensitive to the RSV inhibitor in HEp-2 cells. The emitted fluorescence signal can be analyzed easily and quantitatively, which suggests it is a potential tool for high-throughput screening RSV replication inhibitor in vitro if the optimal parameters are determined. Moreover, compared to other two rRSVs encoding RFP or Luc, rRSV-EGFP seems more competent to be used in such assay, albeit necessary to further investigate the underlying mechanisms.
